# Metformin may reduce oral cancer risk in patients with type 2 diabetes

**DOI:** 10.18632/oncotarget.6626

**Published:** 2015-12-16

**Authors:** Chin-Hsiao Tseng

**Affiliations:** ^1^ Department of Internal Medicine, National Taiwan University College of Medicine, Taipei, Taiwan; ^2^ Division of Endocrinology and Metabolism, Department of Internal Medicine, National Taiwan University Hospital, Taipei, Taiwan; ^3^ Division of Environmental Health and Occupational Medicine of the National Health Research Institutes, Zhunan, Taiwan

**Keywords:** oral cancer, diabetes mellitus, metformin, Taiwan

## Abstract

**Background:**

Whether metformin use may affect the risk of oral cancer required further investigation.

**Methods:**

The reimbursement database of the National Health Insurance in Taiwan was used. Patients with type 2 diabetes mellitus at an onset age of 25-74 years during 1999-2005 and newly treated with either metformin (*n* = 288198, “ever users of metformin”) or other antidiabetic drugs (*n* = 16263, “never users of metformin”) were followed for at least 6 months for oral cancer until December 31, 2011. The treatment effect of metformin (for ever versus never users, and for tertiles of cumulative duration of therapy) was estimated by Cox regression adjusted for propensity score (PS) or incorporated with the inverse probability of treatment weighting (IPTW) using PS.

**Results:**

The respective numbers of incident oral cancer in ever users and never users were 1273 (0.44%) and 119 (0.73%), with respective incidences of 92.7 and 163.6 per 100,000 person-years. The overall hazard ratios (95% confidence intervals) suggested a significantly lower risk [0.584 (0.483-0.707) for PS-adjusted model, and 0.562 (0.465-0.678) for IPTW model]. In tertile analyses, the PS-adjusted hazard ratios (95% confidence intervals) for the first (<21.5 months), second (21.5-45.9 months) and third (>45.9 months) tertile of cumulative duration were 1.403 (1.152-1.708), 0.557 (0.453-0.684) and 0.152 (0.119-0.194), respectively; and were 1.244 (1.024-1.511), 0.526 (0.429-0.645) and 0.138 (0.108-0.176), respectively, for IPTW.

**Conclusions:**

Metformin may significantly reduce the risk of oral cancer, especially when the cumulative duration is more than 21.5 months.

## INTRODUCTION

Metformin exerts anticancer effects in various cancer cell types including the breast [1], endometrium, colon, thyroid and esophagus [2], pancreas [3, 4], stomach [5] and prostate [6]. Epidemiological studies also suggested that metformin use in patients with type 2 diabetes mellitus is associated with a reduced risk of colon cancer [7], bladder cancer [8], breast cancer [9], prostate cancer [10], thyroid cancer [11], endometrial cancer [12] and ovarian cancer [13]. However, whether metformin use can reduce the risk of oral cancer in patients with diabetes remains to be confirmed.

Some recent studies suggested that metformin may inhibit the growth of human head and neck squamous cell carcinoma in cancer cell lines [14]. However, epidemiological studies evaluating the effect of metformin on oral cancer are still rare. A recent meta-analysis [15] suggested that only 3 studies are available from the literature: 2 conducted in the USA evaluating the effect of metformin on the survival of patients with head and neck cancer [16, 17] and 1 from Taiwan comparing the incidence of head and neck cancer in patients with diabetes who used and did not use metformin [18]. Metformin users in patients with diabetes were found to have a significantly 34% lower risk of head and neck cancer (adjusted hazard ratio 0.66, 95% confidence interval 0.55-0.79) [18] and its use was associated with an improvement in the overall survival of patients with head and neck cancer [16, 17]. However, in the study conducted in Taiwan, when head and neck cancer was subcategorized into several categories, it was noted that the reduced risk of oral cancer among metformin users was not statistically significant. The adjusted hazard ratio (95% confidence interval) was 0.81 (0.63-1.03) [18].

Therefore, there is only one study investigating the incidence of oral cancer with regards to metformin use in patients with diabetes, which did not fully support a beneficial effect of metformin [18]. Additionally, dose-response relationship was not evaluated in this previous study. The purpose of the present study was to further evaluate whether metformin use in Taiwanese patients with type 2 diabetes mellitus could be associated with the risk of oral cancer by using the reimbursement databases of the National Health Insurance (NHI). Specifically, the present study tried to address the dose-response relationship by using the tertile cutoffs of cumulative duration of metformin therapy, and to investigate whether the combination use with other antidiabetic drugs would affect the results. Furthermore, to solve the potential problem of “prevalent user bias” [19, 20], newly diagnosed diabetic patients and incident users of metformin were recruited. To reduce the potential risk of “immortal time bias” (the initial period of follow-up during which the outcome can not occur) [19, 21], patients included into the study should have been prescribed antidiabetic drugs for at least two times, and those who were followed for a short period of time (i.e., <180 days) were excluded from analyses. To avoid the potential confounding from the differences in baseline characteristics associated with treatment allocation in non-random observational studies, Cox regression models were created either adjusted for propensity score (PS) or incorporated with the inverse probability of treatment weighting (IPTW) using PS [22]. Analyses were conducted both in an original sample derived from the NHI database and in a matched-pair sample derived from the original sample to examine the consistency of the findings.

## RESULTS

**Figure 1:**
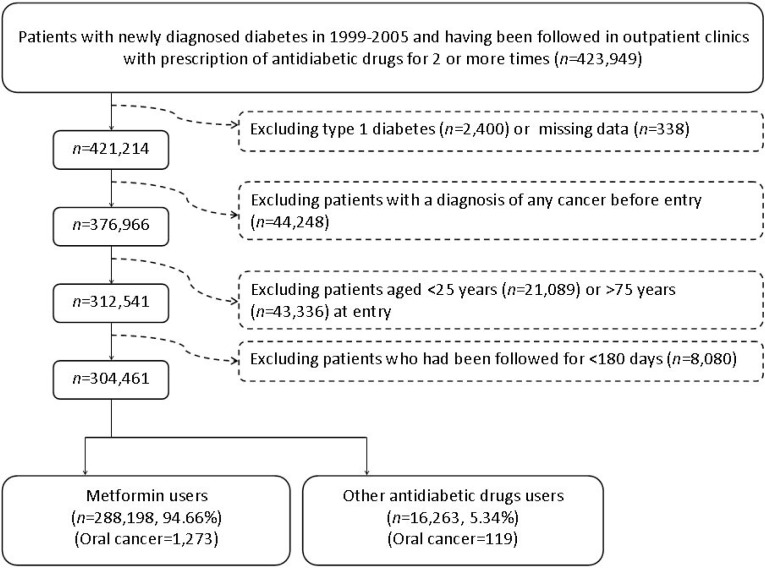
Flowchart showing the procedure in selecting the original sample into the study

There were 16263 never users and 288198 ever users in the original sample (Figure 1). In the original sample, all baseline characteristics (defined at the start of follow-up) of the two groups differed significantly, except for pioglitazone. Ever users were characterized by younger age, less males, higher proportions of eye disease, dyslipidemia, peripheral arterial disease and tobacco abuse, lower proportions of hypertension, nephropathy, stroke, ischemic heart disease, chronic obstructive pulmonary disease and alcohol-related diagnoses, higher proportions of use of rosiglitazone, statin and non-steroidal anti-inflammatory drugs excluding aspirin, but lower proportions of using other antidiabetic medications, angiotensin converting enzyme inhibitor/angiotensin receptor blocker and aspirin (Table 1). It is evident that the baseline characteristics between never users and ever users of metformin were more comparable in the matched sample. Only 5 variables remained significantly different between the two groups, i.e., age, eye disease, sulfonylurea, acarbose and insulin. While examining the standardized differences, 10 out of the 22 variables had values >10% in the original sample, but only sulfonylurea and insulin had values >10% in the matched sample. Actually when covariates were defined at the time of censor, only insulin would have a standardized difference >10% (i.e., −10.41, data for other baseline characteristics are not shown). These findings suggested that results derived from the matched sample would be less likely influenced by residual confounding from the differences in the baseline characteristics.

**Table 1:** Comparison of baseline characteristics between metformin never users and ever users in the original sample and in the propensity score matched sample

Variable	Original sample						Matched sample					
	Never users (n = 16263)		Ever users (n = 288198)		*P*	SD	Never users (n = 16263)		Ever users (n = 16263)		*P*	SD
	n	%	n	%			n	%	n	%		
**Demographic data**												
Age (years)	59.1±10.4		56.6±10.2		<0.0001	−25.14	59.1±10.4		59.4±9.7		0.0197	3.54
Sex (men)	9332	57.4	155199	53.9	<0.0001	−7.31	9332	57.4	9437	58.0	0.2386	0.98
**Major comorbidities**												
Hypertension	11995	73.8	198483	68.9	<0.0001	−11.31	11995	73.8	12033	74.0	0.6315	0.98
Dyslipidemia	9855	60.6	197488	68.5	<0.0001	17.23	9855	60.6	9690	59.6	0.0617	−1.56
**Diabetes-related complications**												
Nephropathy	4139	25.5	46223	16.0	<0.0001	−25.22	4139	25.5	4123	25.4	0.8385	−1.01
Eye disease	1529	9.4	41653	14.5	<0.0001	15.66	1529	9.4	1341	8.3	0.0002	−4.72
Stroke	4021	24.7	54814	19.0	<0.0001	−14.73	4021	24.7	3947	24.3	0.3401	−0.90
IHD	6218	38.2	98033	34.0	<0.0001	−9.33	6218	38.2	6256	38.5	0.6648	0.67
PAD	2516	15.5	45915	15.9	<0.0001	1.20	2516	15.5	2505	15.4	0.8659	−0.32
**Antidiabetic drugs**												
Sulfonylurea	11832	72.8	189914	65.9	<0.0001	−11.51	11832	72.8	12560	77.2	<0.0001	11.65
Meglitinide	1338	8.2	10353	3.6	<0.0001	−20.99	1338	8.2	1245	7.7	0.0565	−1.87
Acarbose	1835	11.3	14531	5.0	<0.0001	−22.46	1835	11.3	1718	10.6	0.0376	−4.03
Insulin	1351	8.3	6100	2.1	<0.0001	−29.42	1351	8.3	990	6.1	<0.0001	−10.71
Pioglitazone	403	2.5	7024	2.4	0.7428	0.31	403	2.5	435	2.7	0.2627	0.51
Rosiglitazone	483	3.0	12961	4.5	<0.0001	8.43	483	3.0	441	2.7	0.1610	−1.98
**Potential risk factors of oral cancer**												
COPD	6521	40.1	110809	38.5	<0.0001	−3.84	6521	40.1	6599	40.6	0.3780	1.19
Tobacco abuse	266	1.6	5915	2.1	0.0002	3.19	266	1.6	247	1.5	0.3978	−0.89
Alcohol-related diagnoses	1037	6.4	15451	5.4	<0.0001	−4.77	1037	6.4	1059	6.5	0.6193	0.21
**Medications that may affect cancer risk**												
ACEI/ARB	9609	59.1	163730	56.8	<0.0001	−5.07	9609	59.1	9660	59.4	0.5650	0.74
Statin	6438	39.6	127216	44.1	<0.0001	9.44	6438	39.6	6354	39.1	0.3403	−0.82
Aspirin	7672	47.2	133390	46.3	0.0267	−2.16	7672	47.2	7560	46.5	0.2133	−1.04
NSAID	16180	99.5	287188	99.7	0.0009	2.70	16180	99.5	16186	99.5	0.6344	0.90

Age is expressed as mean ± standard deviation

Abbreviations

SD: standardized difference, IHD: ischemic heart disease, PAD: peripheral arterial disease, COPD: chronic obstructive pulmonary disease, ACEI/ARB: angiotensin converting enzyme inhibitor/angiotensin receptor blocker, NSAID: non-steroidal anti-inflammatory drugs (excluding aspirin)

Table 2 shows the incidences of oral cancer by metformin exposure in the original sample and the hazard ratios comparing metformin exposed to unexposed patients in the original sample and the matched sample, respectively. Only models derived from covariates defined at the start of follow-up are shown in Table 2. The results derived from models with covariates defined at censor are basically similar to the respective models shown in Table 2 and are not shown here. The respective number of incident oral cancer for ever users and never users was 1273 (0.44%) and 119 (0.73%), with respective incidence of 92.7 and 163.6 per 100,000 person-years (Table 2). When evaluating the distribution of the incident cases of oral cancer by the tertiles of cumulative duration of metformin therapy, there was a trend of decreasing incidence with longer duration of exposure (Table 2). The overall hazard ratios (95% confidence intervals) showed a significantly lower risk of oral cancer associated with metformin use in both the PS-adjusted and the IPTW models in either the original sample or the matched sample. When analyzed by the tertiles of cumulative duration of metformin therapy, a reduced risk was observed for the second and third tertiles in all models. A significantly increased risk was observed for the first tertile in the original sample, but the risk was neutral in the first tertile when the matched sample was analyzed.

**Table 2:** Incidences of oral cancer by metformin exposure in the original sample and the hazard ratios comparing metformin exposed to unexposed patients in the original sample and the matched sample

Original sample								Matched sample				
Metformin use	*n/N*	Person-years	Incidence rate(per 100,000 person-years)	PS-adjusted model		IPTW model		*n/N*	PS-adjusted model		IPTW model	
				HR (95% CI)	*P*	HR (95% CI)	*P*		HR (95% CI)	*P*	HR (95% CI)	*P*
Never users	119/16263	72760.7	163.6	1.000		1.000		119/16263	1.000		1.000	
Ever users	1273/288198	1373325.0	92.7	0.584 (0.483-0.707)	<0.0001	0.562 (0.465-0.678)	<0.0001	77/16263	0.592 (0.444-0.789)	0.0003	0.602 (0.452-0.802)	0.0005
**Tertiles of cumulative duration of metformin therapy (months)**												
Never users	119/16263	72760.7	163.6	1.000		1.000		119/16263	1.000		1.000	
<21.5	715/95463	344951.9	207.3	1.403 (1.152-1.708)	0.0008	1.244 (1.024-1.511)	0.0281	35/5287	1.176 (0.806-1.717)	0.4000	1.127 (0.772-1.645)	0.5359
21.5-45.9	418/94666	471050.1	88.7	0.557 (0.453-0.684)	<0.0001	0.526 (0.429-0.645)	<0.0001	31/5307	0.693 (0.466-1.029)	0.0689	0.703 (0.473-1.043)	0.0802
>45.9	140/98069	557322.9	25.1	0.152 (0.119-0.194)	<0.0001	0.138 (0.108-0.176)	<0.0001	11/5669	0.197 (0.106-0.366)	<0.0001	0.205 (0.110-0.380)	<0.0001

*n*: incident cases of oral cancer, *N*: cases followed, HR: hazard ratio, CI: confidence intervals

PS: propensity score created from variables in Table 1

IPTW: Cox regression model incorporated with the inverse probability of treatment weighting (IPTW) using PS

The models shown in the table were created with covariates defined at the start of follow-up. Additional models created with covariates defined at censor were not much different from the respective models shown in the table.

Table 3 shows the hazard ratios for oral cancer comparing different subgroups of metformin exposure with and without other antidiabetic drugs to a referent group who had never used metformin. It was noted that while compared to never users of metformin, metformin users with or without the use of other antidiabetic drugs consistently showed a lower risk of oral cancer in all models. Patients who used only metformin seemed to have a much lower risk than those who might also have been using other antidiabetic drugs.

**Table 3:** Hazard ratios for oral cancer in different subgroups of metformin exposure with or without other antidiabetic drugs in comparison to a referent group who had never used metformin in the original sample

Different subgroups of metformin use	*n* / *N*	PS-adjusted model			IPTW model		
		HR	95% CI	*P*	HR	95% CI	*P*
**Classification I**							
Never users	119 / 16263	1.000			1.000		
Metformin only	265 / 79327	0.486	(0.391-0.603)	<0.0001	0.466	(0.376-0.579)	<0.0001
Metformin as the first OAD with add-on of other OADs, but without insulin	418 / 83933	0.631	(0.514-0.775)	<0.0001	0.594	(0.485-0.728)	<0.0001
Metformin as add-on to other OADs, but without insulin	565 / 118838	0.624	(0.512-0.762)	<0.0001	0.594	(0.487-0.724)	<0.0001
Metformin with insulin (with or without other OADs)	25 / 6100	0.617	(0.400-0.951)	0.0286	0.571	(0.371-0.878)	0.0108
**Classification II**							
Never users	119 / 16263	1.000			1.000		
Metformin only	265 / 79327	0.486	(0.391-0.603)	<0.0001	0.466	(0.376-0.579)	<0.0001
Metformin as the first OAD with add-on of other OADs and/or insulin	430 / 86458	0.633	(0.515-0.777)	<0.0001	0.595	(0.486-0.729)	<0.0001
Metformin as add-on to other OADs and/or insulin	578 / 122413	0.623	(0.510-0.760)	<0.0001	0.592	(0.486-0.721)	<0.0001

*n*: incident cases of oral cancer, *N*: cases followed, HR: hazard ratio, CI: confidence intervals, OAD: oral antidiabetic drug

PS: propensity score created from variables in Table 1

IPTW: Cox regression model incorporated with the inverse probability of treatment weighting (IPTW) using PS

Models shown in the table are created from the original sample with covariates defined at the start of follow-up. Additional models created with covariates defined at censor were not much different from the respective models shown in the table

## DISCUSSION

The findings of this observational study suggested that metformin use in patients with type 2 diabetes mellitus was associated with a significantly lower risk of oral cancer. This was not only observed in the overall analyses comparing ever users to never users, but also in the second and third tertiles of cumulative duration of metformin therapy (Table 2). Although a significantly higher risk was observed in patients who used metformin for less than 21.5 months in the first tertile in the original sample, this was no more observed in the analyses of the matched sample (Table 2). The combination use of other antidiabetic drugs might slightly attenuate the protective effect of metformin, but this did not totally abrogate the metformin effect either when it was used as an initial treatment or as an add-on to other oral antidiabetic drugs or to insulin (Table 3).

The present study added to the literature by showing a dose-response risk reduction of oral cancer among metformin users (Table 2). Because oral cancer is increasing dramatically in the general population in many countries including Taiwan [23] and diabetes is in an epidemic status all over the world, this study not only provided evidence for the use of metformin as a first-line antidiabetic treatment in terms of oral cancer prevention, its use as an adjuvant anticancer treatment to oral cancer is worthy of further investigation, taking into account the improvement of overall survival among metformin users with head and neck cancer [16, 17].

Several randomized clinical trials are being conducted to evaluate the potential usefulness of metformin together with other chemotherapeutic agents on some solid cancers like the breast, endometrial, prostate, and lung cancer [24]. However, clinical trial focusing specifically on the investigation of the effect of metfomin on oral cancer is still lacking. This study provided an important clue for an in-depth investigation of metformin on the prevention and treatment of oral cancer.

In the previous study by Yen et al., a significantly 34% lower risk of head and neck cancer was observed in the diabetic patients who used metformin [18]. However, they did not observe a significantly reduced risk of oral cancer associated with metformin use when some categories of site-specific head and neck cancer were evaluated [18]. Despite a lack of statistical significance, the adjusted hazard ratio of 0.81 (95% confidence interval: 0.63-1.03) still favored a lower risk of oral cancer in metformin users [18]. Some reasons might explain the lack of statistical significance in the study by Yen et al. [18]. First, the sample sizes of metformin users and cases of oral cancer were much smaller in this previous study and this could lead to a lack of statistical power. Second, as noted in the present study, the risk of oral cancer was actually increased in the first tertile of cumulative duration of metformin therapy of less than 21.5 months in the original sample (Table 2). If the previous study included more metformin users with a short duration of use and did not consider the potential imbalance in baseline characteristics between metformin users and non-users, the estimated hazard ratio might be substantially biased toward the null. Third, the lack of investigating a dose-response relationship might have concealed much of the information in the previous study.

The significantly higher risk of oral cancer observed in the first tertile of cumulative duration of metformin therapy in the original sample (Table 2) might be due to residual confounding from the different baseline characteristics between metformin ever users and never users (Table 1), because this was no more observed in the analyses of the more balanced matched sample (Table 2). Additionally, early users of metformin might have a higher risk of oral cancer which was carried over from the diet control/lifestyle modification period to the early phase of metformin therapy.

Oral cancer is closely associated with smoking, alcohol drinking and betel nut chewing [25, 26]. Due to the lack of such information in the databases, we could only use surrogate diagnoses of chronic obstructive pulmonary disease, tobacco abuse and alcohol-related diagnoses for adjustment. Because betel nut chewing is highly correlated with smoking [27, 28], adjustment for surrogate diagnoses for smoking might also have partially adjusted for the effect of betel nut chewing. Although analyses of the standardized differences did not suggest potential confounding from these variables in either the original sample or the matched sample (Table 1), residual confounding could not be excluded because none of these lifestyle risk factors had been actually measured. On the other hand, a confounder should theoretically be correlated with both the exposure (metformin use) and the outcome (oral cancer), and it should not be an intermediate between exposure and outcome [29], there is little reason to believe that these factors can be determinants for metformin use. Taken together, the residual confounding from these lifestyle factors might be minimal.

Human papillomavirus has also been identified as an important risk factor of oral cancer [30], especially in women [31]. Because we did not have such information for analysis, it was not possible to exclude the confounding effect of this viral infection.

The use of angiotensin converting enzyme inhibitor/angiotensin receptor blocker [32], statin [33], aspirin [34] and non-steroidal anti-inflammatory drugs [34] have been shown to affect cancer risk in some studies and they are commonly used in patients with type 2 diabetes mellitus. Although metformin never users and ever users might have different probability of using these drugs in the original sample (Table 1), they did not exert important confounding on the protective effect of metformin on oral cancer based on the following reasons. First, they have been adjusted for as potential confounders in all analyses (Tables 2 and 3) and the standardized differences did not suggest potential confounding from these covariates in the original sample (Table 1). Second, these variables did not distribute differently between metformin never users and ever users in the matched sample (Table 1), and the analyses in the matched sample gave similar conclusion (Table 2).

The mechanisms for a reduced risk of oral cancer associated with metformin use remains to be explored. Chronic inflammation has been increasingly recognized as a key component of tumor progression in the oral cavity [35]. Patients with diabetes suffer from a significantly higher risk of periodontitis [36, 37], and thus may have a higher risk of oral cancer [23]. Metformin use may reduce inflammation in patients with diabetes either through the improvement of metabolic disturbances such as hyperglycemia, insulin resistance and dyslipidemia or through its inhibition of the proinflammatory cancer-promoting nuclear factor κB and STAT3 pathways [38, 39]. Additionally, metformin may inhibit tumorigenesis by inhibiting mammalian target of rapamycin (mTOR) through the activation of 5′-adenosine monophosphate-activated protein kinase (AMPK) [38]. Recent studies also suggested that metformin may exert an immune-mediated antitumor effect by increasing the number of CD8^+^ tumor-infiltrating lymphocytes [40], and suppress the growth of head and neck squamous cell carcinoma cell lines via global inhibition of protein translation [14]. Metformin impairs one-carbon metabolism and acts like an antifolate drug [41], and suppresses viral replication in hepatitis B [42] and C [43] infection (though whether similar effect can be observed in human papillomavirus infection is not known). Therefore, potential mechanisms linking a reduced risk of oral cancer with the use of metformin may be through its improvement of metabolic parameters, inhibition of chronic inflammation, suppression of mTOR via AMPK activation, modulation of immunity, inhibition of protein translation, impairment of one-carbon metabolism and suppression of viral replication.

This study has several strengths. The databases included all claims records on outpatient visits, emergency department visits and hospital admission, and we caught the diagnoses from all sources. Cancer is considered a severe morbidity by the NHI and most medical co-payments can be waived. Furthermore, there is a low drug cost-sharing required by the NHI and patients with certain conditions, such as those with a low-income household, veterans or patients with prescription refills for chronic disease, are exempted from the drug cost-sharing. Therefore the detection rate of oral cancer would not tend to differ among different social classes. The use of medical records also reduced the potential bias related to self-reporting.

The study limitations included a lack of actual measurement data for confounders such as smoking, alcohol drinking, betel nut chewing, family history, lifestyle, diet, and genetic parameters. In addition, we did not have biochemical data to evaluate their impact. Another limitation is the lack of information on the pathology, grading and staging of oral cancer. Because squamous cell carcinoma represents approximately 95% of all cases of oral cancer in Taiwan [44], the findings of the present study should better be applied to squamous cell carcinoma.

In summary, this study is probably the first to show that metformin use among Taiwanese patients with type 2 diabetes mellitus may significantly reduce the risk of oral cancer, especially when it has been used for more than 21.5 months. This protective effect of metformin is not affected by the use of other antidiabetic drugs and is independent of some medications that may affect cancer risk, such as angiotensin converting enzyme inhibitor/angiotensin receptor blocker, statin, aspirin and non-steroidal anti-inflammatory drugs. However, future confirmation with appropriate consideration of potential confounders such as smoking, alcohol drinking, betel nut chewing and human papillomavirus infection is mandatory.

## MATERIALS AND METHODS

The NHI is a compulsory and universal system of health insurance implemented in Taiwan since March 1995. The NHI covers >99% of Taiwan residents and has contracts with >98% of the hospitals nationwide. Computerized and standard claim documents must be submitted to the Bureau of NHI for reimbursement by the contracted medical institutes.

The NHI reimbursement databases have been handled by the National Health Research Institutes (NHRI) and can be used for academic researches if approved by an ethical review board and the NHRI. The databases contain detailed records of every visit of each patient (including outpatient visits, emergency department visits and hospital admission) and include principal and secondary diagnostic codes, prescription orders, and claimed expenses. This study was approved with an approval number 99274.

Individual identification information was scrambled for the protection of privacy. Diabetes was coded 250.XX and oral cancer 140, 141, 143, 144, 145, 146, 148, and 149, based on the *International Classification of Diseases, Ninth Revision, Clinical Modification* (ICD-9-CM).

Figure 1 shows the procedures in recruiting a cohort of patients with newly diagnosed type 2 diabetes mellitus at an onset age of 25-74 years during the period from 1999 to 2005 into the study (original sample). To assure that diabetes was first diagnosed after 1999, patients who had a diagnosis of diabetes mellitus during 1996-1998 were excluded. Patients should have been followed in the outpatient clinic with prescription of antidiabetic drugs for 2 or more times (*n* = 423949). In Taiwan, patients with type 1 diabetes can be issued a so-called “Severe Morbidity Card” after a certified diagnosis and they are waived of much of the co-payment. Patients who held a Severe Morbidity Card certifying they had type 1 diabetes were also excluded (*n* = 2400). A total of 338 patients were excluded because of missing data. Patients who had been diagnosed as having any cancer before entry were excluded (*n* = 44248). Patients aged <25 (*n* = 21089) or >75 (*n* = 43336) were not included into the analyses. Patients who had been followed up for <180 days (*n* = 8080) were also excluded.

Cumulative duration (months) of metformin use was calculated from the reimbursement databases and tertiles of cumulative duration were used for analyses. Demographic data of age and sex and factors that might be correlated with metformin use, diabetes severity or cancer risk were considered as potential confounders [32-34, 45-47]. These included 1) major comorbidities associated with diabetes mellitus: hypertension (ICD-9-CM code: 401-405) and dyslipidemia (272.0-272.4); 2) diabetes-related complications: nephropathy (580-589), eye disease (250.5, 362.0, 369, 366.41 and 365.44), stroke (430-438), ischemic heart disease (410-414), and peripheral arterial disease (250.7, 785.4, 443.81 and 440-448); 3) antidiabetic drugs: sulfonylurea, meglitinide, acarbose, insulin, pioglitazone and rosiglitazone; 4) potential risk factors of oral cancer: chronic obstructive pulmonary disease (a surrogate for smoking; 490-496), tobacco abuse (305.1, 649.0, 989.84) and alcohol-related diagnoses (291, 303, 535.3, 571.0-571.3, 980.0); and 5) medications that may affect cancer risk: angiotensin converting enzyme inhibitor/angiotensin receptor blocker [32], statin [33], aspirin [34] and non-steroidal anti-inflammatory drugs (excluding aspirin) [34]. Baseline characteristics defined at the start of follow-up between never users and ever users were compared by Student's *t* test for age and by Chi-square test for other variables. The accuracy of disease diagnoses in the NHI database has been studied previously. Agreements between claim data and medical records are moderate to substantial, with Kappa values ranged from 0.55 to 0.86 [48].

The incidence density of oral cancer was calculated for never users and ever users and for different subgroups of exposure to metformin. The numerator of the incidence was the number of patients with incident oral cancer during follow-up, and the denominator was the person-years of follow-up. Follow-up started on the first day of the use of antidiabetic drugs and ended on December 31, 2011, at the time of a new diagnosis of oral cancer, or on the date of death or the last reimbursement record.

Logistic regression was used to create PS from the baseline characteristics as shown in Table 1. The treatment effect was estimated either by Cox regression with adjustment for PS (PS-adjusted models) or incorporated with the inverse probability of treatment weighting (IPTW models) using PS without trimming [22]. Hazard ratios were estimated for ever versus never users, and for each tertile of cumulative duration of metformin therapy compared to never users as referent.

In consideration that the baseline characteristics were imbalanced between metformin ever users and never users, additional analyses were conducted by using a 1:1 matched-pair sample based on 8 digits of PS according to the methods described by Parsons (matched sample) [49]. Because the metformin ever users outnumbered never users in the original sample, the case number for each group in the matched sample was based on the case number of never users in the original sample (i.e., *n* = 16263 in each group).

To further examine whether the use of other antidiabetic drugs might exert an impact on the association between metformin use and oral cancer risk, additional analyses were conducted by categorizing metformin users into different subgroups and hazard ratios were estimated by using never users of metformin as the referent group. In classification I, users of metformin were divided into: 1) metformin only; 2) metformin as the first oral antidiabetic drug with add-on of other oral antidiabetic drugs, but without insulin; 3) metformin as add-on to other oral antidiabetic drugs, but without insulin; and 4) metformin with insulin (with or without other oral antidiabetic drugs). In classification II, metformin users were divided into: 1) metformin only; 2) metformin as the first oral antidiabetic drug with add-on of other oral antidiabetic drugs and/or insulin; and 3) metformin as add-on to other oral antidiabetic drugs and/or insulin.

The covariates in the above analyses were defined at the start of follow-up. To further examine whether the findings might be consistent, all of the above models were also conducted with the covariates defined during the whole observation period until the time of censor.

Although IPTW approach has been used to obtain unbiased estimates, this would not be valid if residual systematic differences in baseline characteristics exist [50]. Austin and Stuart proposed a quantitative method as a formal test for balance diagnostics based on the calculation of standardized difference [50]. Although no consensus has been reached for the value of standardized difference to indicate confounding, a value of >10% for a variable might indicate meaningful imbalance with potential confounding [50]. To examine whether residual systematic differences in the covariates might exist, the standardized differences for all covariates were calculated for the IPTW models in the original sample and the matched sample using the methods described by Austin and Stuart [50].

Analyses were conducted using SAS statistical software, version 9.3 (SAS Institute, Cary, NC). *P* < 0.05 was considered statistically significant.
